# Artemisinin attenuates type 2 diabetic cardiomyopathy in rats through modulation of AGE-RAGE/HMGB-1 signaling pathway

**DOI:** 10.1038/s41598-023-37678-w

**Published:** 2023-07-08

**Authors:** Eman A. E. Farrag, Maha O. Hammad, Sally M. Safwat, Shereen Hamed, Doaa Hellal

**Affiliations:** 1 Department of Clinical Pharmacology, Faculty of Medicine, Mansoura University, Mansoura, Egypt; 2 Department of Medical Biochemistry and Molecular Biology, Faculty of Medicine, Mansoura University, Mansoura, Egypt; 3 Department of Physiology, Faculty of Medicine, Mansoura University, Mansoura, Egypt; 4 Department of Medical Histology, Faculty of Medicine, Mansoura University, Mansoura, Egypt

**Keywords:** Cardiology, Endocrinology, Medical research, Molecular medicine

## Abstract

Diabetes mellitus is a common metabolic disorder. About two-thirds of diabetic patients develop diabetic cardiomyopathy (DCM), which becomes a challenging issue as it severely threatens the patient’s life. Hyperglycemia and the resulting advanced glycated end products (AGE) and their receptor (RAGE)/High Mobility Group Box-1 (HMGB-1) molecular pathway are thought to be key players. Recently, artemisinin (ART) has gained more attention owing to its potent biological activities beyond its antimalarial effect. Herein, we aim to evaluate the effect of ART on DCM and the possible underlying mechanisms. Twenty-four male Sprague–Dawley rats were divided into: control, ART, type 2 diabetic and type 2 diabetic treated with ART groups. At the end of the research, the ECG was recorded, then the heart weight to body weight (HW/BW) ratio, fasting blood glucose, serum insulin and HOMA-IR were evaluated. Cardiac biomarkers (CK-MB and LDH), oxidative stress markers, IL-1β, AGE, RAGE and HMGB-1 expression were also measured. The heart specimens were stained for H&E as well as Masson’s trichrome. DCM induced disturbances in all studied parameters; contrary to this, ART improved these insults. Our study concluded that ART could improve DCM through modulation of the AGE-RAGE/HMGB-1 signaling pathway, with subsequent impacts on oxidative stress, inflammation and fibrosis. ART could therefore be a promising therapy for the management of DCM.

## Introduction

Diabetes mellitus (DM) is a metabolic disease that damages different body organs, causing kidney failure, vision loss, autonomic and peripheral neuropathy, cardiovascular and cerebrovascular diseases^1^. DM is a dominant worldwide health problem whose prevalence is approaching pandemic proportions; there are 451 million people worldwide, and this is predicted to escalate to 693 million by 2045^2^. About two-thirds of elderly diabetic patients exist with myocardial dysfunction, the concept of a definite DM-related cardiomyopathy^3^. Diabetic cardiomyopathy (DCM) ultimately develops congestive heart failure, which threatens the patient’s life, so DCM becomes a challenging issue in the medical field^4^.

Despite extensive studies conducted to understand the pathogenesis of DCM, the exactmechanisms by which hyperglycemia produces DCM are not completely confirmed^5^. It has been assumed that myocardial inflammation, lipid accumulation, oxidative stress, apoptosis and fibrosis are related to the DCM pathogenesis^6^.

Hyperglycemia-induced glycation of proteins, lipids and nucleic acids resulted in the production of advanced glycation end products (AGEs). Increased AGE is one of the most important consequences of hyperglycemia-induced cellular injury. The presence of AGEs in the diabetic heart contributes to the release of reactive oxygen species (ROS), pro-inflammatory cytokines and increased myocardial stiffness via activation of AGE receptors (RAGE)^7^.

High mobility group box 1 protein (HMGB1) is a non-chromosomal nuclear protein that regulates gene transcription and maintains the nucleosome structure. It translocates from nuclear to cytoplasmic organelles and is actively released outside the cells under distress. HMGB-1 plays a crucial role in the progression of diabetic problems and its inhibition might have a potential prospective for treating DCM^8^. AGEs can upregulate the HMGB-1 via increased oxidative stress^9^. Moreover, HMGB-1 intensified AGE mediated signalling pathways via RAGE binding^10^. Although HMGB1 has been implicated in hyperglycaemia-induced heart problems, the fundamental mechanism still remains unclear.

Artemisinin (ART) was discovered by the Chinese professor Youyou Tu in 1972, who was awarded the Clinical Medical Research Award in 2011 and the Nobel Prize in Physiology and Medicine in 2015. Presently, ART-combination therapies have become the standard antimalarial treatment worldwide because ART and its derivatives have the most rapid action on malaria with less adverse effects^11^.

In addition to decades of remarkable progress against malaria, exciting evidence has reinforced that ART-related compounds have great activities beyond antimalarial, such as improvement of cardiovascular disease, anti-virus, anti-neoplastic, anti-inflammatory, antioxidative, and immunosuppressive effects. ART has been reviewed in many human diseases such as rheumatoid, arthritis, systemic lupus erythematosus, and multiple sclerosis^12,13^.

Previous animal studies have found that ART possessed hypoglycemic and anti-hyperlipidemic effects in STZ induced diabetic mice, and even valuable effects on liver and renal functions. Artemisia extract alleviated fatty liver and inflammatory responses in high-fat diet-fed mice. It is proven that ART caused the regeneration of pancreatic beta-cell mass from alpha cells^14^. However, the efficacy of ART for some diabetic complications, such as DCM, remains to be evaluated.

As the treatment of DCM has not yet been undergone, this research will focus on the proposed therapeutic properties of ART in DCM through modulation of the AGE-RAGE/HMGB-1 signaling pathway with subsequent improvement on oxidative stress, inflammation and fibrosis. ART could be an anticipated therapy for the management of DCM.

## Results

### Effect of artemisinin treatment on ECG parameters

The HFD-STZ diabetic rats exhibited a significant elevation in HR (*P* < 0.001), PR interval (*P* < 0.05), R wave amplitude, QRS duration and QT interval (*P* < 0.001) in contrast with the control and ART groups. The diabetic rats treated with ART significantly decreased HR (*P* < 0.001), PR interval, R wave amplitude (*P* < 0.05) and QRS duration (*P* < 0.001) in contrast with the untreated diabetic group. Moreover, the QT interval showed a significant decrease in ART-treated diabetic rats (*P* < 0.001) compared with the untreated diabetic group (Table 1).

**Table 1:** ECG parameters in the study groups.

Parameters	Study groups				Test of significant	*P* value
	Group I (control) (n = 6)	Group II (ART) (n = 6)	Group III (T2DM) (n = 6)	Group IV (T2DM + ART) (n = 6)		
HR	102.83 ± 6.49^A^	105.50 ± 7.09^A^	224.83 ± 19.85^B^	173.83 ± 1.55^C^	F = 133.69	0.001*
PR interval (s)	0.07 (0.04–0.08)^A^	0.06 (0.04–0.08)^A^	0.08 (0.08–0.12)^B^	0.06 (0.06–0.08)^A^	KW = 9.49	0.023**
R wave amplitude (mv)	0.85 ± 0.12^A^	0.85 ± 0.18^A^	1.28 ± 0.20^B^	0.93 ± 0.12^A^	F = 10.005	0.001*
QRS duration (s)	0.04 ± 0.01^A^	0.03 ± 0.01^A^	0.08 ± 0.02^B^	0.04 ± 0.01^A^	F = 20.53	0.001*
QT interval (s)	0.08 ± 0.01^A^	0.08 ± 0.01^A^	0.18 ± 0.02^B^	0.11 ± 0.01^C^	F = 67.28	0.001*

Data are presented as mean ± SD and compared by one-way ANOVA. **Data are presented as median (IQR) and compared by the Kruskal–Wallis H test. Pairwise comparison is displayed as capital letters (similar letters mean a statistically insignificant difference, while different letters mean a statistically significant difference), *P* values are significant if ≤ 0.05. ART, artemisinin; T2DM, type 2 diabetes mellitus; HR, heart rate.

### Effect of artemisinin treatment on HW, BW and HW/BW ratio

The HFD-STZ diabetic group showed a significant increase (*P* ≤ 0.001) in the HW/BW ratio in comparison with the control and ART groups. The ART-treated diabetic group revealed a significant reduction (*P* < 0.001) in the HW/BW ratio in comparison with the HFD-STZ diabetic group (Table 2).

**Table 2:** Heart weight/body weight (mg/gm) and glucose profiles in the study groups.

Parameters	Study groups				F value	*P* value
	Group I (control) (n = 6)	Group II (ART) (n = 6)	Group III (T2DM) (n = 6)	Group IV (T2DM + ART) (n = 6)		
Heart weight (HW) (mg)	692 ± 51^A^	777 ± 45^A,C^	1265 ± 69^B^	863 ± 97^C^	81.77	0.001
Body weight (BW) (gm)	270.8 ± 14.4^A^	264.8 ± 10.6^A^	229 ± 9.9^B^	275.3 ± 10.4^A^	20.20	0.001
HW/BW (mg/gm)	2.57 ± 0.31^A^	2.93 ± 0.08^A^	5.53 ± 0.30^B^	2.92 ± 0.50^A^	101.49	0.001
FBG (mg/dl)	96.67 ± 2.80^A^	89 ± 2.83^A^	396 ± 18.24^B^	169.17 ± 14.29^C^	893.74	0.001
Insulin (pg/ml)	223.83 ± 4.31^A^	238.17 ± 5.56^B^	106.5 ± 6.60^C^	185.50 ± 11.62^D^	366.39	0.001
HOMA-IR	1.54 ± 0.06^A^	1.50 ± 0.03^A^	2.99 ± 0.31^B^	2.22 ± 0.15^C^	95.94	0.001

Data are expressed as mean ± SD. Comparison between the groups is performed with the one-way ANOVA test and followed by the post-hoc Tukey’s test, which is displayed in capital letters (similar letters mean a statistically insignificant difference, while different letters mean a statistically significant difference), *P* values are significant if ≤ 0.05. ART, artemisinin; T2DM, type 2 diabetes mellitus; HW/BW**,** heart weight/body weight; FBG, fasting blood glucose; HOMA-IR, homeostasis model assessment index.

### Effect of artemisinin treatment on glucose profiles

Table 2 shows the glucose profiles of all the study groups. The FBG levels and HOMA-IR of HFD-STZ diabetic rats were significantly higher than those of the control and ART groups (*P* < 0.001). The treatment of diabetic rats with ART significantly (*P* < 0.001) reduced the FBG levels and HOMA-IR compared to the HFD-STZ diabetic non-treated rats.

Serum insulin levels in HFD-STZ diabetic rats were significantly lower than those in the control and ART groups (*P* < 0.001). There was a significant increase in the diabetic + ART group compared to the HFD-STZ diabetic group (*P* < 0.001), but it was still lower than the normal control group.

### Effect of artemisinin treatment on cardiac biomarkers (LDH and CK-MB)

In order to investigate the hyperglycemia-induced cardiomyopathy and the protective effect of Artemisinin, we investigated the serum levels of LDH and CK-MB cardiac biomarkers. Our results illustrated higher levels of LDH and CK-MB in the HFD-STZ diabetic group compared to the non-diabetic control rats (*P* < 0.001). ART had the potential to significantly reverse this change in the diabetic + ART group compared to the diabetic rats (*P* < 0.001) Table 3.

**Table 3:** Biochemical parameters in the study groups.

Parameters	Study groups				F value	*P* value
	Group I (control) (n = 6)	Group II (ART) (n = 6)	Group III (T2DM) (n = 6)	Group IV (T2DM + ART) (n = 6)		
LDH (U/L)	1400 ± 13.04^A^	1414.17 ± 14.29^A^	2235 ± 14.14^B^	1518.33 ± 8.16^C^	410.39	0.001**
CK-MB (ng/ml)	0.36 ± 0.01^A^	0.33 ± 0.01^A^	0.51 ± 0.04^B^	0.36 ± 0.02^A^	66.59	0.001**
MDA (nmol/g)	31.77 ± 1.62^A^	28.75 ± 0.90^A^	81.72 ± 3.50^B^	39.50 ± 1.15^C^	870.23	0.001**
GSH (mmol/mg)	0.95 ± 0.09^A^	0.92 ± 0.06^A^	0.40 ± 0.08^B^	0.76 ± 0.06^C^	72.83	0.001**
IL-1β (pg/mg)	47.87 ± 1.12^A^	50.48 ± 0.60^A^	110.50 ± 2.97^B^	61.45 ± 2.59^C^	481.27	0.001**
AGE (ng/mg)	0.82 ± 0.08^A^	1.03 ± 0.08^A,B^	3.55 ± 0.27^C^	1.22 ± 0.15^B^	371.23	0.001**

Data are expressed as mean ± SD. Comparison between the groups is performed with the one-way ANOVA and followed by the post hoc Tukey’s test, which is displayed in capital letters (similar letters mean statistically insignificant difference, while different letters mean statistically significant difference), P values are significant if ≤ 0.05. ART, artemisinin; T2DM, type2 diabetes mellitus; MDA, malondialdehyde; GSH, reduced glutathione; LDH, lactate dehydrogenase; CK-MB, creatine kinase-MB; AGE, advanced glycation end product; IL-1β, interleukin-1 beta.

### Effect of artemisinin treatment on oxidative stress biomarkers

The cardiac tissue levels of MDA and GSH in the four study groups are demonstrated (Table 3). There was a significant elevation in the HFD-STZ diabetic group compared to the control and ART groups (*P* < 0.001). ART-treated diabetic group, exhibited a significant reduction in tissue MDA in comparison with the HFD-STZ diabetic group (*P* < 0.001).

The hearts of the HFD-STZ diabetic rats displayed diminished GSH content when compared with other groups (*P* < 0.001), which was reversed by ART treatment (*P* < 0.001).

### Effect of artemisinin treatment on IL-1β and AGE in the cardiac tissues

The cardiac tissue contents of IL-1β and AGE are demonstrated in (Table 3). Cardiac tissues of the HFD-STZ diabetic rats displayed increased IL-1β and AGE levels compared with other groups (*P* < 0.001). This finding was inverted in the diabetic + ART group (*P* < 0.001).

### Effect of artemisinin treatment on RAGE mRNA expression

**Figure 1:**
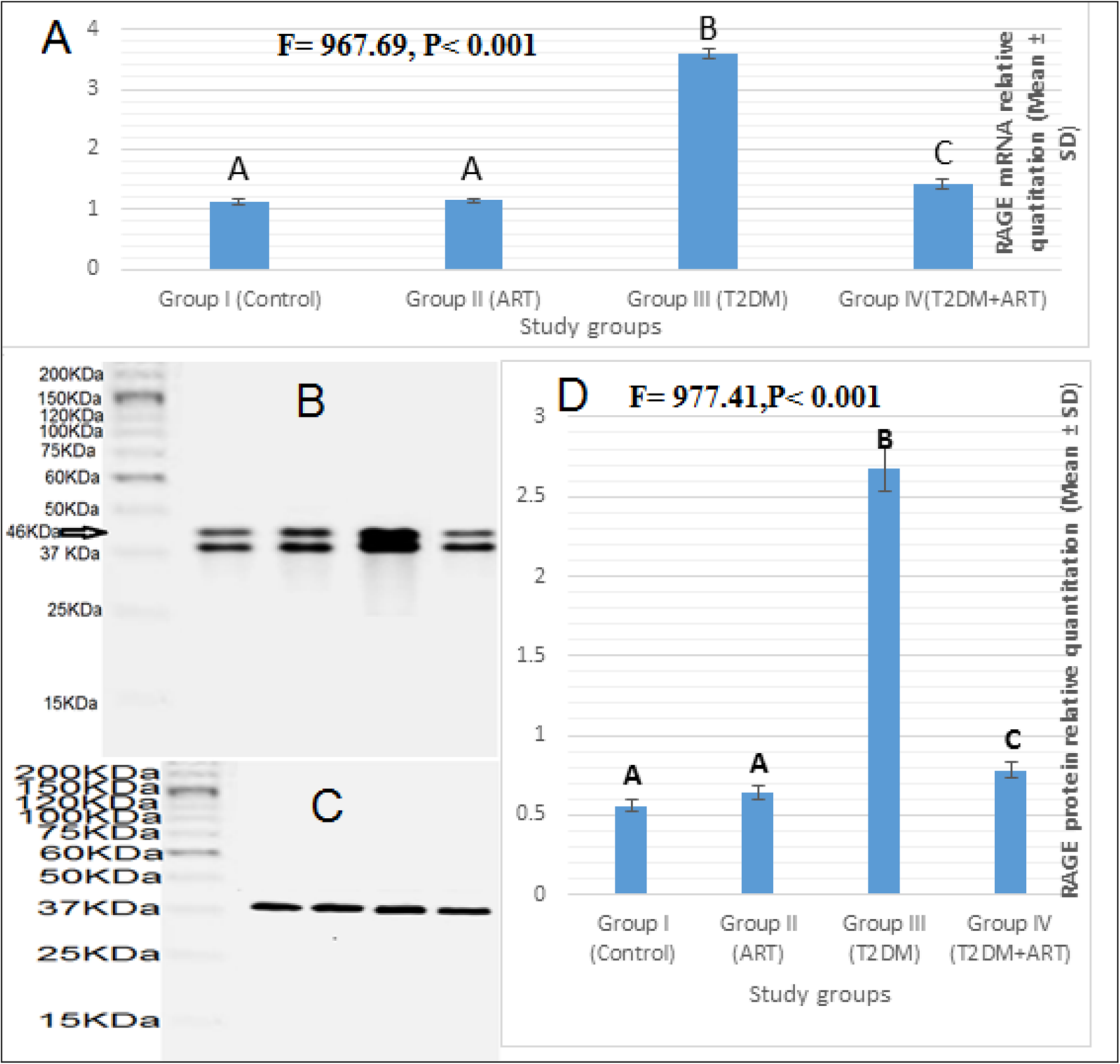
Expression of RAGE in rat cardiac tissues among all study groups (I, II, III, IV). (**A**) *RAGE* mRNA expression as determined by RT-qPCR. (**B**–**D**) RAGE protein expression as determined by western blotting. (**B**) Representative picture for RAGE protein expression by western blotting (molecular weight: 46 kDa). (**C**) β-actin protein bands (molecular weight: 43 kDa). β-actin is selected as an endogenous control. (**D**) *RAGE* protein relative quantitation by western blotting. Data are expressed as mean ± SD. Comparison between the groups is performed with the one-way ANOVA test and followed by the post-hoc Tukey’s test, which is displayed in capital letters (similar letters mean a statistically insignificant difference, while different letters mean a statistically significant difference), with significant *P* values (≤ 0.05). ART, artemisinin; T2DM, type 2 diabetes mellitus.

To confirm the protective effect of ART on the DCM, the relative quantitation in RAGE mRNA expression was detected by qRT-PCR (Fig. 1). There was a significant difference in RAGE mRNA expression between the four groups (*P* < 0.001). Post hoc Tukey adjustment for RAGE mRNA expression showed a significant increase in the HFD-STZ diabetic group compared to the control and ART groups (*P* < 0.001). Moreover, RAGE mRNA expression in the diabetic rats treated with ART was significantly decreased (*P* < 0.001) compared to the HFD-STZ diabetic group.

### Effect of artemisinin treatment on RAGE protein expression

Western blotting was utilized to evaluate the RAGE protein expression in the four groups (Fig. 1). It exhibited a statistically significant difference in RAGE protein between the four groups (*P* < 0.001). Post hoc Tukey adjustment showed a significant increase in RAGE protein in the HFD-STZ diabetic group compared with the other groups (*P* < 0.001). Upon investigating the RAGE protein in the diabetic + ART group, there was a significant decrease in RAGE protein compared to that in the HFD-STZ diabetic group (*P* < 0.001).

### Effect of artemisinin treatment on cardiac histopathology

**Figure 2:**
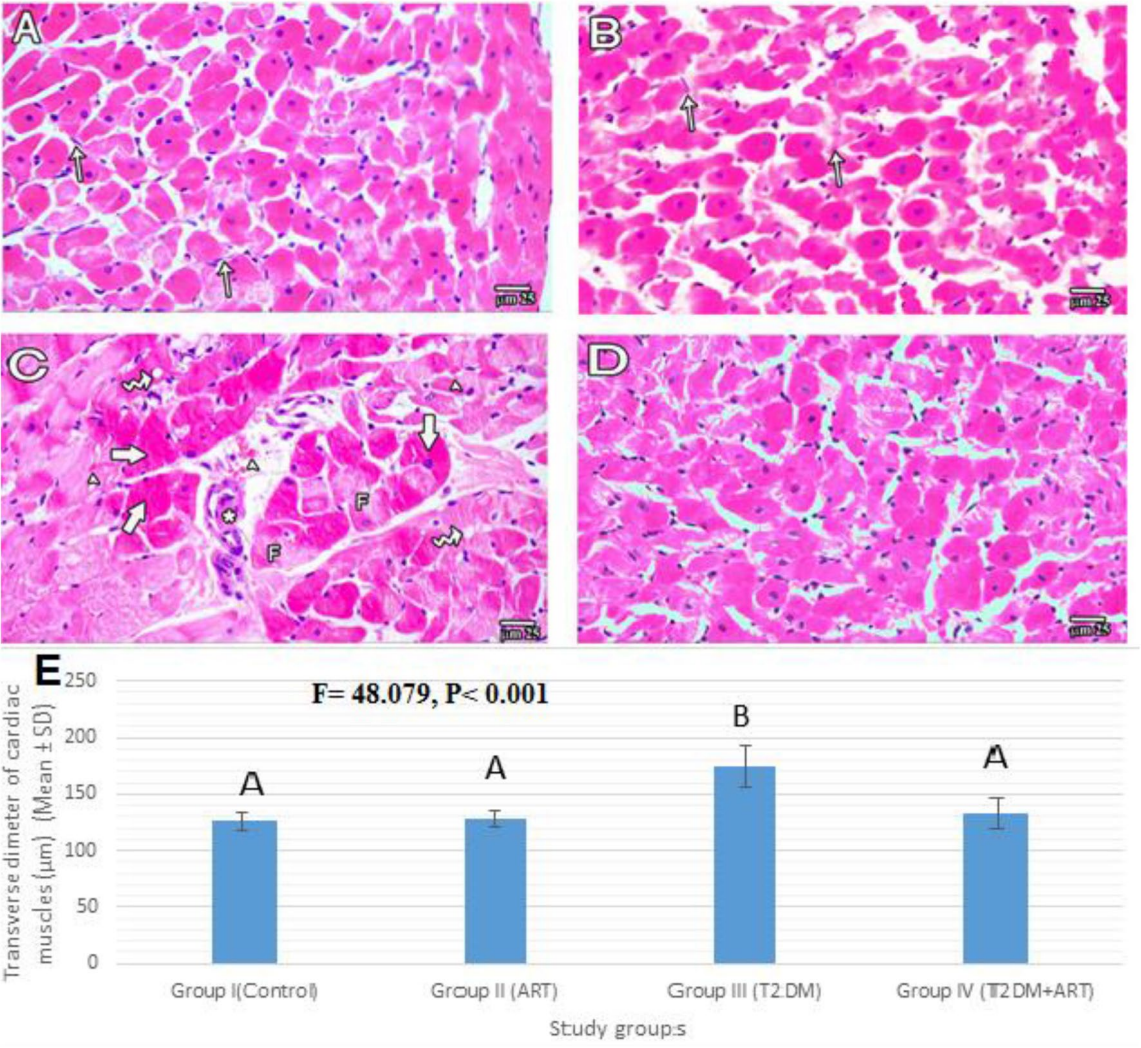
Photomicrographs of H&E stained sections of the myocardium of the left ventricle among all study groups (I, II, III, IV). (**A**,**B**) H&E of Group I (control) and Group II (ART) respectively show transversely cut polyhedral cardiac muscle fibers with mostly central vesicular nuclei and acidophilic cytoplasm with loose connective tissue containing fibroblasts with flat nuclei (arrows). (**C**) Group III (T2DM) shows myocardial cytoplasmic fragmentation (f) and vacuolation (zigzag arrows) and many cardiac muscle fibers of large diameter (thick arrows), some cardiac muscles are replaced by cellular infiltrates (*) with extravasated RBCs. (**D**) Group IV (T2DM + ART) appears more or less similar to Group I (H&E, bar 25 µm). (**E**) Mean of transverse diameters of cardiac muscle fibers (µm) in the study groups. Data are presented as mean ± SD. Comparison between the groups is performed with the one-way ANOVA test and followed by the post-hoc Tukey’s test, which is displayed in capital letters (similar letters mean a statistically insignificant difference, while different letters mean a statistically significant difference). *P* values are significant if ≤ 0.05. ART, artemisinin; T2DM, type2 diabetes mellitus.

Routine histological examination of the myocardium revealed that normal control and ART groups revealed polygonal cardiac muscle fibers with acidophilic cytoplasm and a central vesicular nucleus in most fibers. The cardiac muscle fibers were surrounded by loose connective tissue containing fibroblasts with oval nuclei. The HFD-STZ diabetic rats showed degenerative changes in the form of myocardial cytoplasmic fragmentation and vacuolation. Additionally, many cardiac muscle fibers of large diameter and some cardiac muscles were replaced by cellular infiltrates. Group IV (T2DM + ART group) appeared almost similar to the normal control group (Fig. 2).

The HFD-STZ diabetic rats displayed a significant increase in the transverse diameter of cardiac muscles compared with the control groups (Fig. 2E) (*P* < 0.001). The treatment of diabetic rats with ART revealed a significant decrease in the transverse diameter of cardiac muscles (*P* < 0.001) compared with the untreated diabetic group, while ART + diabetic rats exhibited a non-significant change compared with the control groups.

**Figure 3:**
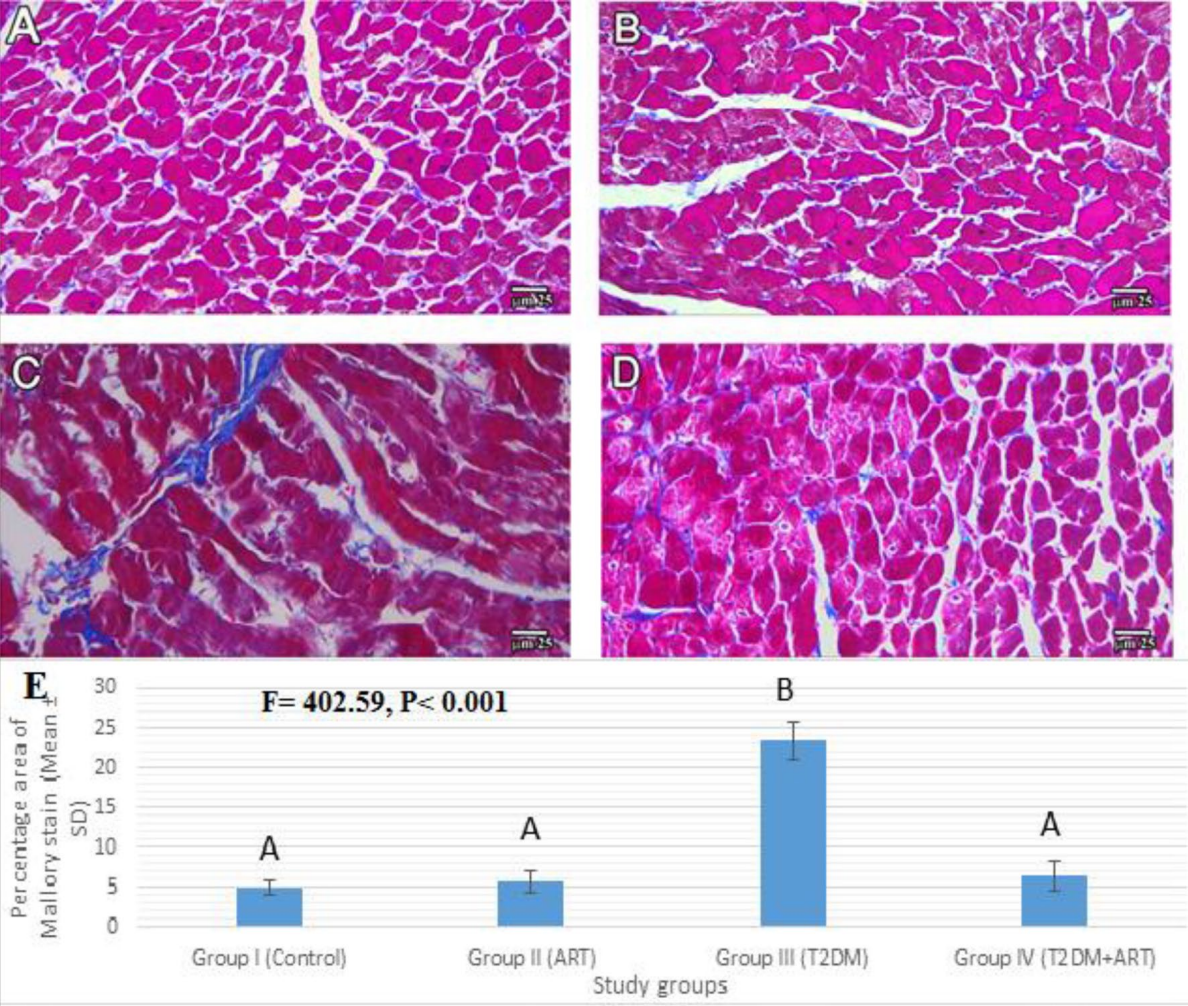
Photomicrographs of Masson’s trichrome stained sections of the myocardium of the left ventricle among all study groups (I, II, III, IV). (**A**,**B**) Group I (control) and Group II (ART) respectively show fine interstitial collagen fibers. (**C**) Group III (T2DM) shows obviously increased deposition of collagen fibers. (**D**) Group IV (T2DM + ART group) appears more or less similar to group I (Masson’s trichrome staining, bar 25 µm). (**E**) Percentage area of Masson’s trichrome stain in the study groups. Data are presented as mean ± SD. Comparison between the groups is performed with the one-way ANOVA test and followed by the post-hoc Tukey’s test, which is displayed in capital letters (similar letters mean a statistically insignificant difference, while different letters mean a statistically significant difference), *P* values are significant if ≤ 0.05. ART, artemisinin; T2DM, type 2 diabetes mellitus.

To evaluate the degree of collagen deposition, we stained sections of the myocardium in various groups with Masson’s trichrome stain. The HFD-STZ diabetic rats showed a statistically significant increase in the percentage area of Masson’s trichrome stain compared with the normal control and ART groups (*P* < 0.001). The percentage area of Masson’s trichrome stain in the diabetic + ART group showed a significant decrease compared to that in the diabetic untreated group (*P* < 0.001) (Fig. 3).

### Effect of artemisinin treatment on the cardiac HMGB-1

**Figure 4:**
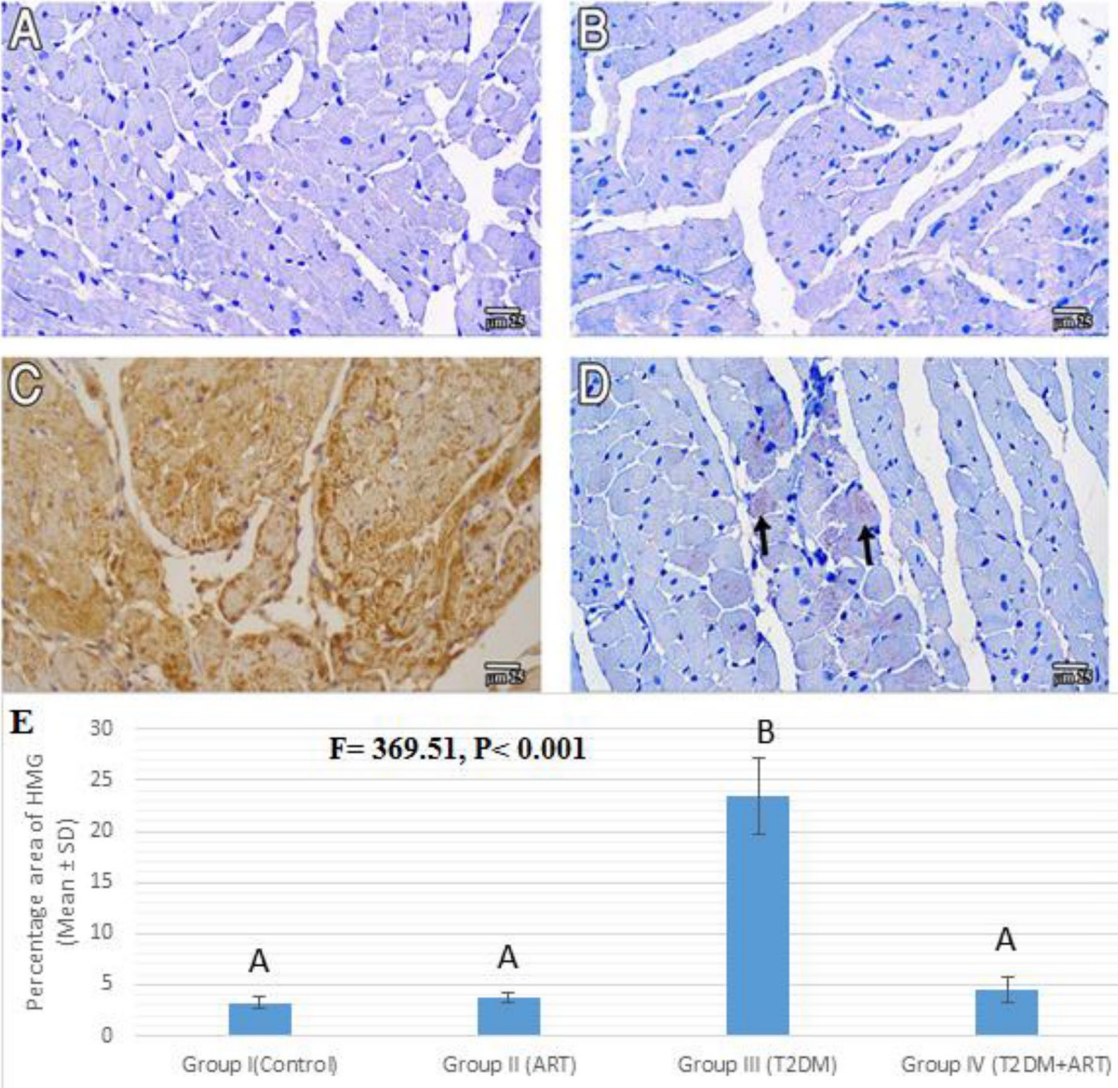
Photomicrographs of HMGB-1 immunostained sections of the myocardium of left ventricle among all study groups (I, II, III, IV). (**A**,**B**) Group I (control), Group II (ART) respectively show mostly negative reaction. (**C**) Group III (T2DM) shows very strong positive nuclear and cytoplasmic immune brown reactions in many cardiac muscle fibers. (**D**) Group IV (T2DM + ART) shows mostly negative immune reaction with the exception of some areas that show weak cytoplasmic reaction in some cardiac muscle fibers (black arrows) (HMGB-1 immunostaining, bar 25 µm). (**E**) Percentage area of immune stain in the study groups. Data are expressed as mean ± SD. Comparison between the groups is performed with the one-way ANOVA test and followed by the post-hoc Tukey’s test, which is displayed in capital letters (similar letters mean a statistically insignificant difference, while different letters mean a statistically significant difference). *P* values are significant if ≤ 0.05. ART, artemisinin; T2DM, type 2 diabetes mellitus.

The HFD-STZ diabetic rats revealed a significant increase in the percentage of immunostained areas for HMGB-1 (*P* < 0.001) compared with the control and ART groups. The treatment of diabetic rats with ART revealed a significant decrease in the percentage of the immunostained areas for HMGB-1 (*P* < 0.001) compared with the untreated diabetic group, while the ART treatment of diabetic rats exhibited a non-significant change compared with the control and ART groups (Fig. 4).

## Discussion

DCM is a diabetes-associated complication, which is known as the most essential cause of raised morbidity and mortality among diabetic patients^15^. Chemical antidiabetics have many side effects and are not effective in managing diabetic complications; therefore, alternative therapies have been involved in the studies of diabetes and its complications^16^. One of the therapies is ART, which has ultimate benefits for the cardiovascular system, but its effects on diabetes-related cardiac complications are still in the introductory stage. Accordingly, more confirmation is needed to establish its role in DCM^17^.

Our data based on the HFD-STZ induced DCM rat model showed that ART treatment for 8 weeks at a dose of 75 mg/kg/day can alleviate cardiac ECG findings, cardiac biomarkers, inflammatory mediators, oxidative biomarkers, myocardial fibrosis and myocyte disarray associated with DCM.

This study demonstrated increased heart rates, PR interval, QT interval, R wave amplitude and QRS duration in HFD-STZ diabetic rats; signifying the electrical change effect of HFD-STZ on ECG findings. This result agrees with the previous findings of Youssef et al.^18^. Left ventricular hypertrophy with myocardial fibrosis is a typical sign of diabetic cardiomyopathy^19^. These stimulate the sympathetic tone, which results in increased activity of the sarcoplasmic reticulum by excessive stimulation of β-receptors^20^, leading to increased Ca^++^ release, which could explain the QT interval prolongation^21^. The development of DCM leads to the formation of the left ventricular bundle block and dysfunction in ventricular conductivity, resulting in prolongation of the QRS complex^22^. ART administration to diabetic rats’ alleviated ECG changes that appeared in untreated diabetic rats. The improved cardiac function with ART application probably resulted from the improvement in coronary artery endothelium-dependent relaxation and increased myocardial blood supply^23^.

HFD-STZ diabetic rats showed significant increases in the HW/BW ratio. This result is in agreement with the result of Jia et al.^24^, who reported that hyperglycemia prompts cardiac hypertrophy via cardiac IR and metabolic disorders that increase mitochondrial dysfunction, AGEs and inflammation. The loss in body weight is due to an increase in muscle protein catabolism, glycogenolysis, lipolysis and polyuria^25^. ART-treated diabetic rats exhibited a significant reduction in the HW/BW ratio. Xiong et al.^26^ demonstrated that ART can attenuate cardiac hypertrophy through its anti-inflammatory properties.

Regarding the glucose profiles, this study showed a significant elevation in serum glucose, HOMA-IR and a significant decline in serum insulin in the HFD-STZ diabetic group. These results agree with studies performed by Feng et al.^27^ and Zaheri et al.^28^. Insulin resistance is a condition in which cells fail to respond to the normal insulin actions, resulting in hyperglycemia due to impaired glucose utilization by the cells. IR along with decreased insulin secretion, results in T2DM^29^. Moreover, a high fat diet is a responsible factor in developing IR^30^. IR is an important cause in the development of DCM^31^. It plays a fundamental role in physiopathology in the initiation and progression of in vivo metabolic disorders^32^.

ART-treated diabetic rats revealed a significant reduction in glucose levels and HOMA-IR and an increase in insulin levels. It has been reported that ART treatment attenuates diabetic hyperglycemia by elevating insulin secretion, which has been observed in rats, mice and human islets^33^. ART treatment could upregulate insulin and the insulin-like growth factor binding protein-1^34^. Additionally, several researches have demonstrated that ART can increase insulin sensitivity and attenuate IR^35,36^.

In the present study, diabetes-related cardiac damage was demonstrated by increased circulating levels of CK-MB and LDH. These results are consistent with those of other studies^37–39^. Our findings showed that diabetic rats treated with ART significantly decreased serum CK-MB and LDH. Wang et al.^40^ revealed that ART administration lowered CK-MB and LDH in myocardial ischemia/reperfusion injury^39^. ART can inhibit cardiac damage by inhibiting the expression of the inflammatory factor IL-1β and decreasing the infiltration of macrophages.

Regarding oxidative stress, the untreated HFD-STZ diabetic rats in the current research exhibited a significant elevation in the cardiac MDA, while GSH was significantly diminished, these results are concomitant with those of Al-Rasheed et al.^41^. Hyperglycemia causes overproduction of mitochondrial superoxide and oxidative stress (OS), which can damage vascular endothelial cells and internal organs, mostly the organs with plentiful vessels, such as the heart^42^. Tissue depletion of GSH and increased MDA levels lead to OS and consequent tissue damage^43,44^. Moreover, our study reported that ART treatment significantly decreased MDA and increased GSH levels. Zhang et al.^45^ reported the positive effects of ART on MDA and GSH levels. ART could regulate OS to control cellular processes; however, detailed understanding of the molecular mechanisms remains to be explored^13^.

The pro-inflammatory markers are elevated in T2DM patients^46^. Among the pro-inflammatory cytokines, IL-1β was found to be particularly important because it is the most elevated circulating pro-inflammatory factor in diabetic patients^47^. In this study, we demonstrated a significant increase in IL-1β in the untreated diabetic group. This is in harmony with the results of Yapislar et al.^48^. Excessive IL-1β is produced by pancreatic β-cells under circumstances of hyperglycemia^49^. Our results demonstrated that ART administration significantly decreased IL-1β. This is in harmony with the results of Fu et al.^36^, which revealed the ability of ART to stimulate AMP-activated protein kinase (AMPK) activity and suppress inflammatory factor expression; thus inverse the pathological state. They predicted that the anti-inflammatory effects of ART have a causal association with diminished IR.

In the present study, we found that the untreated diabetic rats displayed a significant elevation in both AGE and RAGE in cardiac tissues, which is in line with a previous observation^50^. Moreover, this study demonstrated that ART treatment can decrease AGE levels and down-regulate RAGE at the mRNA and protein levels. Chen et al.^51^ demonstrated that ART may play a protective role against cardiovascular complications of type-1 diabetes via suppressing the expression of proteins in the RAGE/NF-κB signaling pathway and decreasing inflammatory factors.

AGE accumulation is a main factor in the development of diabetic complications because AGEs are irreversibly accumulated in the body, dependent on the degree of blood sugar and duration^52^. AGEs accelerate the expression of RAGEs^53^. RAGE has been found to interact with AGEs as their receptors and has been implicated in a range of diabetic complications^54^. AGEs-RAGE axis has a great role in the pathogenesis of DCM through inducing endothelial dysfunction, changing calcium handling/contractility and inducing inflammatory, oxidative stress and fibrotic reactions in the myocardium^55^.

The AGE plays a role in inducing the translocation and release of HMGB-1 from the nucleus to the cytoplasm, which promotes oxidative stress binding to the RAGE and induces an inflammatory response via several signaling pathways^56^. Moreover, HMGB-1 stimulates AGE-induced pro-inflammatory cytokine expression^57^. Thus, RAGE transduced the signals of both AGEs and HMGB-1^9^.

In this study, we found that HMGB-1 expression and translocation were increased in the cardiomyocytes of the HFD-STZ diabetes untreated rats, which conforms to the previous observation of Wang et al.^58^ who proposed that HMGB-1 is related to diabetes-associated myocardial dysfunction and inhibition of HMGB-1 might have potential prospectives in the treatment of DCM. Interestingly, our study reported that ART treatment significantly decreased HMGB1 in cardiomyocytes compared with the diabetic untreated rats. This result is attributed to the effect of ART on the AGE-RAGE axis and subsequent modulation of HMGB1 translocation and release. This observation is in agreement with the results of Kim et al.^59^, which demonstrated that administration of ART reduced hepatic HMGB-1 expression in HFD-fed mice.

The histological analysis of heart tissue from the HFD-STZ diabetic rats showed significant myofiber disruption and increased collagen deposition as detected by H&E and Masson’s trichrome staining, which is in agreement with that of Wang et al.^60^. Additionally, diabetic rats showed an increased percentage of fibrosis and transverse diameter of myocardial fibers with a raggedy ridged appearance, which is a significant histopathological characteristic of dilated cardiomyopathy^61^.

The rationale for increased fibrosis in DCM may be due to increased AGE, which induces alterations in the mechanical properties of the extracellular matrix by elevating resistance to connective tissue enzymatic proteolysis and stimulating the crosslinking of collagens and laminins^62^. Moreover, AGEs bind to RAGE to prompt ROS and inflammatory gene expression, which increases matrix proteins via the activation of mitogen-activated protein kinase and Janus kinase in cardiac tissues^63^. Moreover, previous studies have demonstrated that translocated HGMB-1 induced the expression of collagens and profibrogenic factors, and induces fibroblast activation in vitro and in vivo^8^. Therefore, hyperglycemia and the associated activation of AGEs-RAGE/HMGB-1 signaling observed in diabetic rats are important contributors to myocardial fibrosis.

In this study, the ART treatment of diabetic rats alleviated cardiomyocyte fibrosis mainly by modulating the AGE-RAGE/HMGB-1 signaling pathway. Previously, in vitro and in vivo studies have shown anti-fibrotic effects of ART by inhibiting epithelial-mesenchymal transformation^64^.

Finally, our data point to a modifying effect of ART on HFD-STZ induced DCM. ART ameliorates functional, biochemical, molecular and morphological changes of DCM. ART attenuates the AGE-RAGE/HMGB-1 signaling pathway with subsequent modulation of oxidative stress, cardiomyocyte inflammation and fibrosis. Therefore, ART could be a promising therapy for the management of DCM.

## Methods

### Animals

The protocol for animal use was permitted by the Institutional Research Board (IRB), Faculty of Medicine, Mansoura University (Protocol number: R.21.06.1367). Adult male Sprague–Dawley rats (n = 24; weight = 160 ± 20 g) were obtained from the Medical Experimental Research Centre (MERC), Faculty of Medicine, Mansoura University. The required diet and tap water were supplied. Rats were exposed to a 12-h light/dark cycle and a room temperature of 18–22 °C. The rats were euthanized by cervical dislocation. All methods involving rats were carried out in accordance with standard conventional guidelines and regulations. The experiment was conducted in accordance with ARRIVE guidelines.

### Experimental design and induction of type 2 diabetes

Rats were randomly divided into a control non-diabetic group (n = 6), ART non-diabetic group (n = 6) and a diabetic group (n = 12). Type 2 DM rat model was established as described by Zhang et al.^57^. The diabetic group was fed a high fat diet (HFD), comprising 34.5% fat, 17.5% protein and 48% carbohydrate. After 4 weeks of HFD, the diabetic group was given a single intraperitoneal injection of streptozotocin (Sigma, St. Louis, MO; 27.5 mg/kg i.p. in 0.1 mol/L citrate buffer, pH 4.5). One week after the administration of streptozotocin, rats with blood glucose levels > 200 mg/dl in two sequential evaluations were considered diabetic rats. Then, diabetic rats were subdivided into two groups: a diabetic (n = 6) and a diabetic + ART (n = 6), which received ART with a dose of (75 mg/kg/day)^44^ by gavage for 8 weeks. The control non-diabetic and ART non-diabetic groups received ordinary chow and intraperitoneal injections of citrate buffer containing the same volume of streptozotocin.

At the end of the experiment, rats in all groups were weighed, fasted for 12 h and anaesthetized via intraperitoneal injection of a mixture of ketamine (70–84 mg/kg) and xylazine (9 mg/kg). Then, electrocardiography (ECG) was recorded. Fasting blood samples were collected via cardiac puncture in tubes with no EDTA. Blood samples were left to clot for 10 min, centrifuged for serum separation and stored at − 20 °C. Finally, the hearts were separated, dried, weighed and preserved for further analysis.

### ECG monitoring

Anesthetized rats were placed supine and needle electrodes were implanted subcutaneously into the four limbs. ECG leads I, II, III, aVR, aVL, aVF were recorded via a single-channel digital electrocardiograph (MSC-2001, Medical System International Corporation NY) with a paper speed of 50 mm/s and sensitivity of 20 mm/mV (X2). Analysis of the recorded traces was completed as regards heart rate, P wave duration and amplitude, PR interval, R wave duration and amplitude and QT interval^65^.

### Heart weight to body weight (HW/BW) ratio

Cardiac hypertrophy was detected by identifying the ratio of heart dry weight to total body weight (HW/BW).

### Colorimetric assay of fasting blood glucose (FBG) and cardiac biomarkers (CK-MB and LDH)

FBG was estimated colorimetrically using a test reagent kit (Biodiagnostic, Egypt) after being oxidized enzymatically to yield a violet quinoneimine. While cardiac biomarkers (CK-MB and LDH) were measured by kinetic colorimetric assay using special kits (Biomed, Egypt) in accordance with the manufacturer’s instructions and via the Erba CHEM-7 apparatus (ERBA Diagnostics, India)^66^.

### Enzyme-linked immunosorbent assay (ELISA) of Insulin

Insulin levels were assessed via a rat insulin ELISA kit (Cloud-Clone Corp., China) based on the competitive inhibition enzyme immunoassay technique. Absorbance was measured at 450 nm using a ChroMate 4300-microplate reader (Awareness Technologies, USA)^67^.

### Homeostasis model assessment index (HOMA-IR)

Insulin resistance was assessed by HOMA-IR = insulin (μU/ml) × glucose (mg/dl) ÷ 405^68^.

### Colorimetric assay of tissue malondialdehyde (MDA) and reduced glutathione (GSH) levels

Animals’ cardiac tissues were washed and splashed with ice. 10% of the homogenate was prepared in 0.05 M phosphate buffer (pH7) utilizing a polytron homogenizer at 4 °C. The homogenate was centrifuged at 10,000 rpm for 20 min and the clear supernatants were collected and stored on ice. The MDA level was estimated as a marker for lipid peroxidation. MDA reacts with thiobarbituric acid (TBA) and the pink MDA-TBA products were measured at 535 nm colorimetrically according to the manufacturer’s instructions of a commercially available kit (Biodiagnostic, Egypt). GSH level was evaluated using the commercially available kit (Biodiagnostic, Egypt), GSH reduces 5,5-dithiobis (2-nitrobenzoic acid), resulting in a yellow-colored product. The concentration of the yellow product is directly related to the level of GSH and its absorbance can be measured at 405 nm^69^.

### ELISA of AGE and IL-1β in cardiac tissues

The tissue contents of AGE and IL-1β were determined using rat AGE and rat IL-1β ELISA kits (LifeSpan Biosciences, Inc., USA) based on the sandwich principle. The absorbance was assessed spectrophotometrically at 450 nm via a ChroMate 4300-microplate reader (Awareness Technologies, USA)^70^.

### Analyses of mRNA expression by quantitative real-time PCR

Cardiac tissues were collected in RNAlater reagent (500 μl RNAlater/50 mg cardiac tissue sample) (Qiagen, Germany), kept overnight at 4 °C, and then conveyed to − 80 °C to be stored until tissue homogenization. Extraction of total RNA was accomplished from homogenized tissues of all groups via the QIAzol reagent (Qiagen, Germany) in accordance with the constructor’s guidelines, and then quantity and quality of RNA yield were assessed by NanoDrop (Thermo Fisher Scientific, USA) by estimating the absorbance at 260 nm and 280 nm. Reverse transcription of 1 μg RNA into cDNA was performed according to manufacturer’s instructions of the SensiFAST™ cDNA Synthesis Kit (Bioline, UK) using the thermal cycler (Applied Biosystem, USA) in a thermal profile of the following: 10 min at 25 °C for primer annealing, 15 min at 42 °C for reverse transcription, and 5 min at 90 °C for inactivation. Finally, cDNA templates were intensified utilizing a real-time PCR apparatus (Applied Biosystems 7500, USA) in an amplification profile of the following: 2 min at 98 °C followed by 40 cycles of 10 s at 95 °C and 30 s at 60 °C. The amplification reaction contained 10 μl of HERA SYBR green PCR Master Mix (Willowfort, UK), 2 μl of cDNA, 2 μl gene primer (10 pmol/μl) and 6 μl of nuclease-free water. The RAGE primers (*Rattus norvegicus*; PCR amplicon: 150 bp; RefSeq: NM_053336.2): F:5′-AGAAACCGGTGATGAAGGAC-3′ and R:5′-TCGAGTCTGGGTTGTCGTTT-3′; and the GAPDH primers (*Rattus norvegicus*; PCR amplicon: 169 bp; RefSeq: NM_017008.4): F:5′-CCTCGTCTCATAGACAAGATGGT-3′ and R: 5′-GGGTAGAGTCATACTGGAACATG-3′. Primers were designed using Primer3 software (v.4.1.0; http://primer3.ut.ee). After the real-time PCR run, the data were exhibited as Cycle threshold (Ct) for the target gene and control gene. The relative quantitation (RQ) of mRNA expression of the target gene is quantified in accordance with the calculation of 2^−∆∆Ct^ method^71^.

### Western blotting

The total protein extraction was performed via the QIAzol reagent (Qiagen, Germany) in accordance with the constructor’s instructions, then the protein concentration was determined in each sample via a Bradford assay (Bosterbio, Canada). Amounts of 20 μg protein were separated using a 10% sodium dodecyl sulfate (SDS)/polyacrylamide gel (PAGE) and then conveyed to a 0.22 μm nitrocellulose membrane (Abcam, USA) via the Eco-Line Biometra instrument (Gottingen, Germany). The membrane was blocked in tris-buffered saline with Tween-20 (TBS-T) buffer and 3% bovine serum albumin (BSA) at room temperature for 1 h. The membranes were incubated overnight at 4 °C with rabbit monoclonal anti-RAGE (1:1000, ab216329, Abcam, USA) and rabbit polyclonal anti-β-actin (1:1000, ab8227, Abcam, USA). After probing with primary antibodies, membranes were washed thrice (10 min/wash) with TBS-T to remove unbound antibodies and then incubated with appropriate HRP-conjugated with goat anti-rabbit secondary antibodies (1:2000, ab6721, Abcam, USA) for 1 h at room temperature. The protein bands were visualized with a chemiluminescence substrate (Clarity™ Western ECL substrate Bio-Rad, USA), and the signals were captured using a CCD camera-based imager. The protein expression was normalized to the control protein β-actin and band intensity was analyzed via the ChemiDoc MP imager^72^.

### Histopathology analysis

Cardiac tissues were embedded in 10% neutral formaldehyde, consequently dehydrated in rising grades of alcohol, cleared in xylene, fixed in paraffin, and then 4 μm-thick sections were produced. The sections were stained with H&E as a routine histopathological examination and with Masson-Trichrome for evaluation of cardiac tissue fibrosis according to Bancroft and Gamble^73^.

### Morphometric analysis

H/E stained sections were used for assessing the transverse diameter of the cardiac muscle fibers^74^. The percentage area of both collagen fibers stained with Masson’s-trichrome and the immunopositive reaction of HMGB-1 stained sections were evaluated^75^. The stained sections were analyzed and photographed utilizing an Olympus Microscope BX-51 (Olympus) with a digital camera and a computer, using a 40X objective. The resulting images were analyzed on ImageJ 1.47v software (National Institutes of Health, USA). Five slides from every group were set and five random fields from each slide were examined.

### Immunohistochemical staining

Paraffin sections were deparaffinized, rehydrated and boiled in a sodium citrate buffer solution (pH 6.0) at 95 °C for 15 min. Sections were incubated overnight at 4 °C with primary polyclonal antibodies against rabbit HMGB1 (1:200, Bioss Inc, USA), then with HRP-conjugated anti-rabbit antibodies, and finally samples were stained with 3,3-diaminobenzidine. Normal rabbit IgG was substituted for the primary antibody as the negative control; the nuclei were counterstained with hematoxylin. Brown cytoplasmic staining was considered a positive reaction^76^.

### Statistical analysis

The SPSS software (version 25.0, IBM, Chicago, IL, USA) was used for the analysis of the data, which were expressed as the mean ± SD. The one-way ANOVA test was used for statistical analyses of the data obtained followed by the Post-hoc Tukey’s test for multiple comparisons of the group means. *P* values less than 0.05 indicated statistically significant differences.

## Data Availability

The datasets used and/or analyzed during the current are study available from the corresponding author upon reasonable request.

## Supplementary Figures

Supplementary Information.

## References

[1] Solanki ND, Bhavsar SK (2015). An evaluation of the protective role of *Ficus racemosa* Linn. in streptozotocin-induced diabetic neuropathy with neurodegeneration. Indian J. Pharmacol..

[2] Cho N (2018). IDF diabetes atlas: Global estimates of diabetes prevalence for 2017 and projections for 2045. Diabetes Res. Clin. Pract..

[3] Jia G, Whaley-Connell A, Sowers JR (2018). Diabetic cardiomyopathy: A hyperglycaemia- and insulin-resistance-induced heart disease. Diabetologia.

[4] Goyal BR, Solanki N, Goyal RK, Mehta AA (2009). Investigation into the cardiac effects of spironolactone in the experimental model of type 1 diabetes. J. Cardiovasc. Pharmacol..

[5] Liu Q, Wang SD, Cai L (2014). Diabetic cardiomyopathy and its mechanisms: Role of oxidative stress and damage. J. Diabetes Investig..

[6] Falcão-Pires I, Leite-Moreira AF (2012). Diabetic cardiomyopathy: Understanding the molecular and cellular basis to progress in diagnosis and treatment. Heart Fail. Rev..

[7] Al Hroob AM, Abukhalil MH, Hussein OE, Mahmoud AM (2019). Pathophysiological mechanisms of diabetic cardiomyopathy and the therapeutic potential of epigallocatechin-3-gallate. Biomed. Pharmacother..

[8] Wu H (2016). High mobility group Box-1: A missing link between diabetes and its complications. Mediators Inflamm..

[9] Wu H (2016). Diabetes-induced oxidative stress in endothelial progenitor cells may be sustained by a positive feedback loop involving high mobility group box-1. Oxid. Med. Cell. Longev..

[10] Fukami K, Yamagishi SI, Okuda S (2014). Role of AGEs–RAGE system in cardiovascular disease. Curr. Pharm. Des..

[11] Dai YF (2017). The pharmacological activities and mechanisms of artemisinin and its derivatives: A systematic review. Med. Chem. Res..

[12] Shi C, Li H, Yang Y, Hou L (2015). Anti-inflammatory and immunoregulatory functions of artemisinin and its derivatives. Mediators Inflamm..

[13] Xia M, Liu D, Liu Y, Liu H (2020). The therapeutic effect of artemisinin and its derivatives in kidney disease. Front. Pharmacol..

[14] Guo Y (2018). Antidiabetic and antiobesity effects of artemether in db/db mice. BioMed Res. Int..

[15] Chavali V, Tyagi SC, Mishra PK (2013). Predictors and prevention of diabetic cardiomyopathy. Diabetes Metab. Syndr. Obes..

[16] Özdek U, Yıldırım S, Değer Y (2020). The effect of *Diplotaenia turcica* root extract in streptozotocin-induced diabetic rats. Turkish J. Biochem..

[17] Jiang YY, Shui JC, Zhang BX, Chin JW, Yue RS (2020). The potential roles of artemisinin and its derivatives in the treatment of type 2 diabetes mellitus. Front. Pharmacol..

[18] Youssef ME (2022). Electrocardiographic and histopathological characterizations of diabetic cardiomyopathy in rats. Environ. Sci. Pollut. Res..

[19] Luneva EB (2022). Simple predictors for cardiac fibrosis in patients with type 2 diabetes mellitus: The role of circulating biomarkers and pulse wave velocity. J. Clin. Med..

[20] Assis FR (2019). Cardiac sympathectomy for refractory ventricular tachycardia in arrhythmogenic right ventricular cardiomyopathy. Heart Rhythm.

[21] Paavola J (2016). Slowed depolarization and irregular repolarization in catecholaminergic polymorphic ventricular tachycardia: A study from cellular Ca^2+^ transients and action potentials to clinical monophasic action potentials and electrocardiography. EP Eur..

[22] Akgun T, Kalkan S, Tigen MK (2014). Variations of QRS morphology in patients with dilated cardiomyopathy; clinical and prognostic implications. J. Thorac. Cardiovasc. Surg..

[23] Liu X (2021). Artemisinin improves acetylcholine-induced vasodilatation in rats with primary hypertension. Drug Des. Dev. Therapy.

[24] Jia G, Hill M, Sowers J (2018). Diabetic cardiomyopathy: An update of mechanisms contributing to this clinical entity. Circ. Res..

[25] Bolla K, Sri KV, Varalakshmi K (2015). Diabetes mellitus and its prevention. Int. J. Sci. Technol. Res..

[26] Xiong Z (2010). Artemisinin, an anti-malarial agent, inhibits rat cardiac hypertrophy via inhibition of NF-κB signaling. Eur. J. Pharmacol..

[27] Feng XT, Tang SY, Jiang YX, Zhao W (2017). Anti-diabetic effects of Zhuoduqing formula, a Chinese herbal decoction, on a rat model of type 2 diabetes. Afr. J. Tradit. Complement. Altern. Med..

[28] Zaheri Z, Fahremand F, Rezvani ME, Karimollah A, Moradi A (2019). Curcumin exerts beneficial role on insulin resistance through modulation of SOCS3 and Rac-1 pathways in type 2 diabetic rats. J. Funct. Foods..

[29] Moonishaa TM (2017). Evaluation of leptin as a marker of insulin resistance in type 2 diabetes mellitus. Int. J. Appl. Basic Med. Res..

[30] Ohtsubo K, Chen MZ, Olefsky JM, Marth JD (2011). Pathway to diabetes through attenuation of pancreatic beta cell glycosylation and glucose transport. Nat. Med..

[31] Ti Y (2011). TRB3 gene silencing alleviates diabetic cardiomyopathy in a type 2 diabetic rat model. Diabetes.

[32] Takeuchi M, Takino JI, Sakasai-Sakai A, Takata T, Tsutsumi M (2017). Toxic AGE (TAGE) theory for the pathophysiology of the onset/progression of NAFLD and ALD. Nutrients.

[33] Li J (2017). Artemisinins target GABA(A) receptor signaling and impair α cell identity. Cell.

[34] Xiang M, Chen Z, He L, Xiong G, Lu J (2019). Transcription profiling of artemisinin-treated diabetic nephropathy rats using high-throughput sequencing. Life Sci..

[35] Guo Y (2018). Antidiabetic and antiobesity effects of artemether in db/db mice. BioMed Res. Int..

[36] Fu W (2020). Artemether regulates metaflammation to improve glycolipid metabolism in db/db mice. Diabetes Metab. Syndr. Obes..

[37] Al-Rasheed NM, Hasan IH, Al-Amin MA, Al-Ajmi HN, Mahmoud AM (2016). Sitagliptin attenuates cardiomyopathy by modulating the JAK/STAT signaling pathway in experimental diabetic rats. Drug Des Dev. Ther..

[38] Fouda A, El-Aziz A, Mabrouk N (2019). Effects of Arabic gum on cardiomyopathy in a rat model of type II diabetes. Al-Azhar Med. J..

[39] Wang F (2020). Artemisinin suppresses myocardial ischemia–reperfusion injury via NLRP3 inflammasome mechanism. J. Cell Mol. Med..

[40] Gu Y (2012). Artemisinin suppresses sympathetic hyperinnervation following myocardial infarction via anti-inflammatory effects. J. Mol. Histol..

[41] Al-Rasheed NM (2017). Simvastatin ameliorates diabetic cardiomyopathy by attenuating oxidative stress and inflammation in rats. Oxid. Med. Cell. Longev..

[42] Luo J (2020). Allopurinol reduces oxidative stress and activates Nrf2/p62 to attenuate diabetic cardiomyopathy in rats. J. Cell Mol. Med..

[43] Sharifi-Rad M (2020). Lifestyle, oxidative stress, and antioxidants: Back and forth in the pathophysiology of chronic diseases. Front. Physiol..

[44] Ribas V, García-Ruiz C, Fernández-Checa JC (2014). Glutathione and mitochondria. Front. Pharmacol..

[45] Zhang H, Qi S, Song Y, Ling C (2020). Artemisinin attenuates early renal damage on diabetic nephropathy rats through suppressing TGF-β1 regulator and activating the Nrf2 signaling pathway. Life Sci..

[46] Lontchi-Yimagou E, Sobngwi E, Matsha TE, Kengne AP (2013). Diabetes mellitus and inflammation. Curr. Diabetes Rep..

[47] Alfadul H, Sabico S, Al-Daghri NM (2022). The role of interleukin-1β in type 2 diabetes mellitus: A systematic review and meta-analysis. Front. Endocrinol..

[48] Yapislar H (2022). Anti-inflammatory effects of melatonin in rats with induced type 2 diabetes mellitus. Life.

[49] Liu Z (2015). Circulating interleukin-1β promotes endoplasmic reticulum stress-induced myocytes apoptosis in diabetic cardiomyopathy via interleukin-1 receptor-associated kinase-2. Cardiovasc. Diabetol..

[50] Abdelmageed ME, Shehatou GS, Abdelsalam RA, Suddek GM, Salem HA (2019). Cinnamaldehyde ameliorates STZ-induced rat diabetes through modulation of IRS1/PI3K/AKT2 pathway and AGEs/RAGE interaction. Naunyn-Schmiedeb. Arch. Pharmacol..

[51] Chen Y (2021). Role of Artesunate on cardiovascular complications in rats with type 1 diabetes mellitus. BMC Endocr. Disord..

[52] Singh R, Barden A, Mori T, Beilin L (2001). Advanced glycation end-products: A review. Diabetologia.

[53] Rhee SY, Kim YS (2018). The role of advanced glycation end products in diabetic vascular complications. Diabetes Metab. J..

[54] Chen XJ (2019). Advanced glycation end-products induce oxidative stress through the Sirt1/Nrf2 axis by interacting with the receptor of AGEs under diabetic conditions. J. Cell Biochem..

[55] Bodiga VL, Eda SR, Bodiga S (2014). Advanced glycation end products: Role in pathology of diabetic cardiomyopathy. Heart Fail. Rev..

[56] Zhang L (2016). Early administration of trimetazidine attenuates diabetic cardiomyopathy in rats by alleviating fibrosis, reducing apoptosis and enhancing autophagy. J. Transl. Med..

[57] Cheng M (2015). HMGB1 enhances the AGE-induced expression of CTGF and TGF-β via RAGE-dependent signaling in renal tubular epithelial cells. Am. J. Nephrol..

[58] Wang WK (2014). Inhibition of high-mobility group box 1 improves myocardial fibrosis and dysfunction in diabetic cardiomyopathy. Int. J. Cardiol..

[59] Kim KE (2016). Artemisia annua leaf extract attenuates hepatic steatosis and inflammation in high-fat diet-fed mice. J. Med. Food..

[60] Wang Z (2020). Protective effects of AS-IV on diabetic cardiomyopathy by improving myocardial lipid metabolism in rat models of T2DM. Biomed. Pharmacother..

[61] Mitrut R, Stepan AE, Pirici D (2018). Histopathological aspects of the myocardium in dilated cardiomyopathy. Curr Health Sci J..

[62] Lazo M (2015). Soluble receptor for advanced glycation end products and the risk for incident heart failure: The atherosclerosis risk in communities study. Am. Heart J..

[63] Jia G, DeMarco VG, Sowers JR (2016). Insulin resistance and hyperinsulinaemia in diabetic cardiomyopathy. Nat. Rev. Endocrinol..

[64] Zhang Y (2017). Role of artesunate in TGF-β1-induced renal tubular epithelial-mesenchymal transdifferentiation in NRK-52E cells. Mol. Med. Rep..

[65] Ola-Davies OE, Olukole SG (2018). Gallic acid protects against bisphenol A-induced alterations in the cardio-renal system of Wistar rats through the antioxidant defense mechanism. Biomed. Pharmacother..

[66] Sharma R, Kumar A, Srinivasan BP, Chauhan A, Dubey K (2014). Cardioprotective effects of *Ficus religiosa* in neonatal streptozotocin-induced diabetic cardiomyopathy in rats. Biomed. Aging Pathol..

[67] Sharma AK, Srinivasan BP (2009). Triple verses glimepiride plus metformin therapy on cardiovascular risk biomarkers and diabetic cardiomyopathy in insulin resistance type 2 diabetes mellitus rats. Eur. J. Pharm. Sci..

[68] Okita K (2013). Homeostasis model assessment of insulin resistance for evaluating insulin sensitivity in patients with type 2 diabetes on insulin therapy. Endocr. J..

[69] Althunibat OY (2019). Fisetin ameliorates oxidative stress, inflammation and apoptosis in diabetic cardiomyopathy. Life Sci..

[70] Hou J (2016). Mangiferin suppressed advanced glycation end products (AGEs) through NF-κB deactivation and displayed anti-inflammatory effects in streptozotocin and high fat diet-diabetic cardiomyopathy rats. Can. J. Physiol. Pharmacol..

[71] Ganger MT, Dietz GD, Ewing SJ (2017). A common base method for analysis of qPCR data and the application of simple blocking in qPCR experiments. BMC Bioinform..

[72] Liu ZQ, Mahmood T, Yang PC (2014). Western blot: Technique, theory and trouble shooting. N. Am. J. Med. Sci..

[73] Bancroft, J. & Gamble, M. Hematoxylin and eosin, connective tissue and stain, carbohydrates. *Theory and practice in histological techniques. 6th ed. Churchill-Livingstone, Edinburgh*. 121–186 (2008).

[74] Ozlu B (2019). A bioartificial rat heart tissue: Perfusion decellularization and characterization. Int. J. Artif. Organs.

[75] Costa GM (2019). Picrosirius red and masson’s trichrome staining techniques as tools for detection of collagen fibers in the skin of dogs with endocrine dermatopathologies. Ciênc. Anim. Bras..

[76] Wang WK (2014). HMGB 1 mediates hyperglycaemia-induced cardiomyocyte apoptosis via ERK/Ets-1 signaling pathway. J. Cell Mol. Med..

