# Supplementary material for: Artemisinin attenuates type 2 diabetic cardiomyopathy in rats through modulation of AGE-RAGE/HMGB-1 signaling pathway
Source: Sci Rep. 2023 Jul 8;13:11043. doi: 10.1038/s41598-023-37678-w (PMC10329689; doi:10.1038/s41598-023-37678-w)
Supplement: Supplementary file 1 — Supplementary Information. [file 41598_2023_37678_MOESM1_ESM.pptx]

## Slide 1
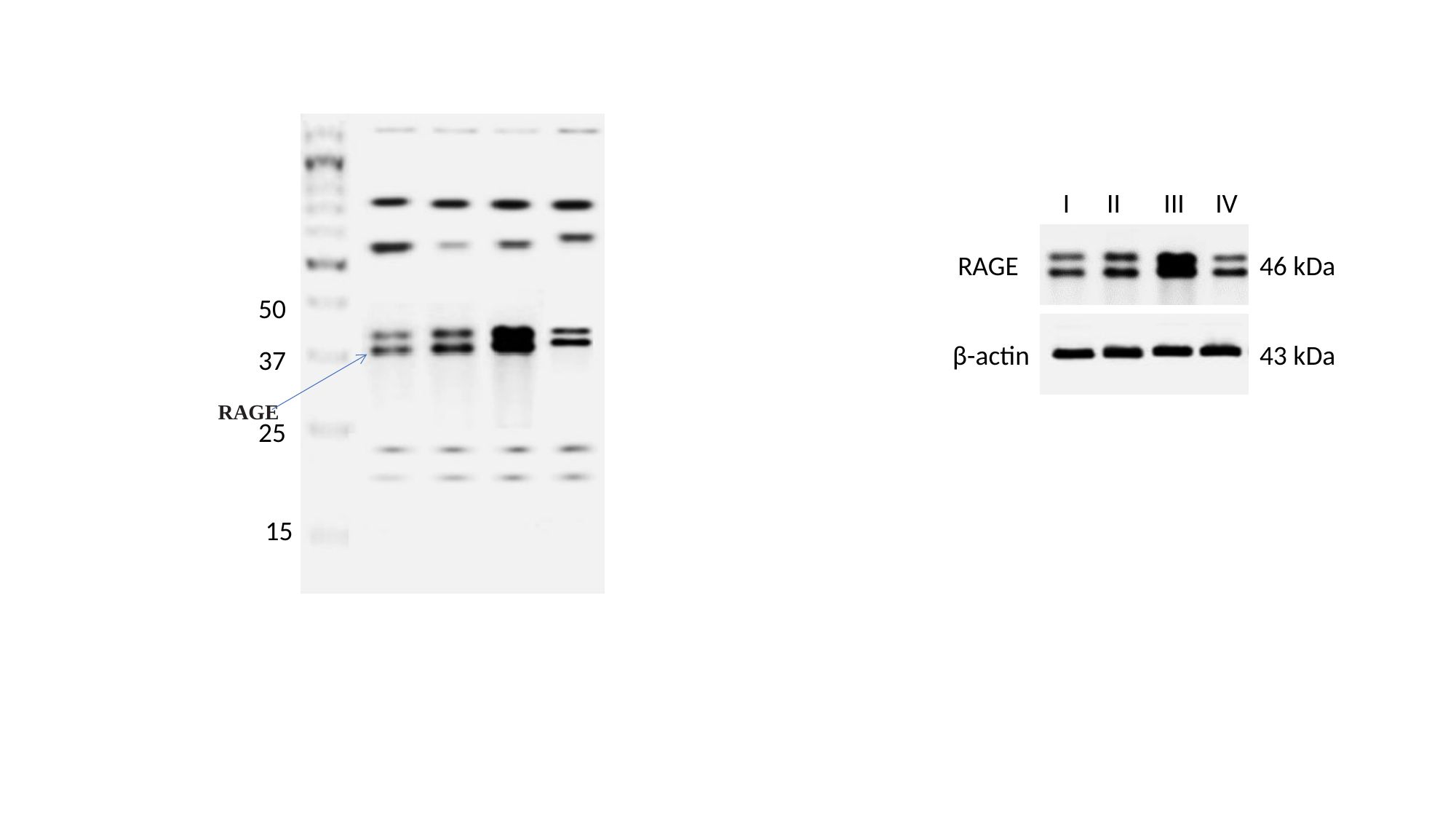

I II III IV
RAGE
46 kDa
43 kDa
β-actin
50
37
RAGE
25
15

## Slide 2
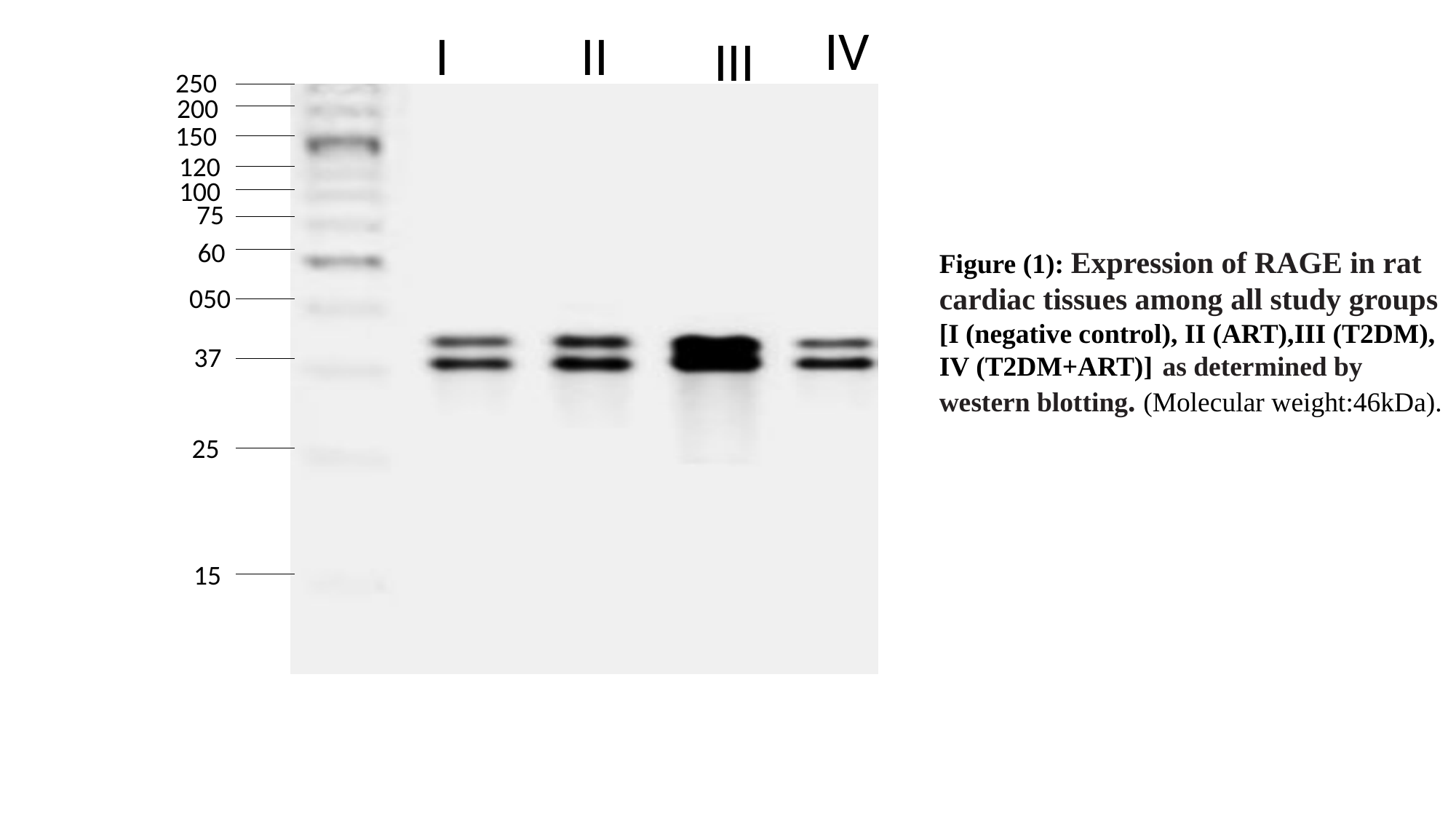

IV
II
I
III
250
200
150
120
100
75
 60
050
37
25
15
Figure (1): Expression of RAGE in rat cardiac tissues among all study groups [I (negative control), II (ART),III (T2DM), IV (T2DM+ART)] as determined by western blotting. (Molecular weight:46kDa).

## Slide 3
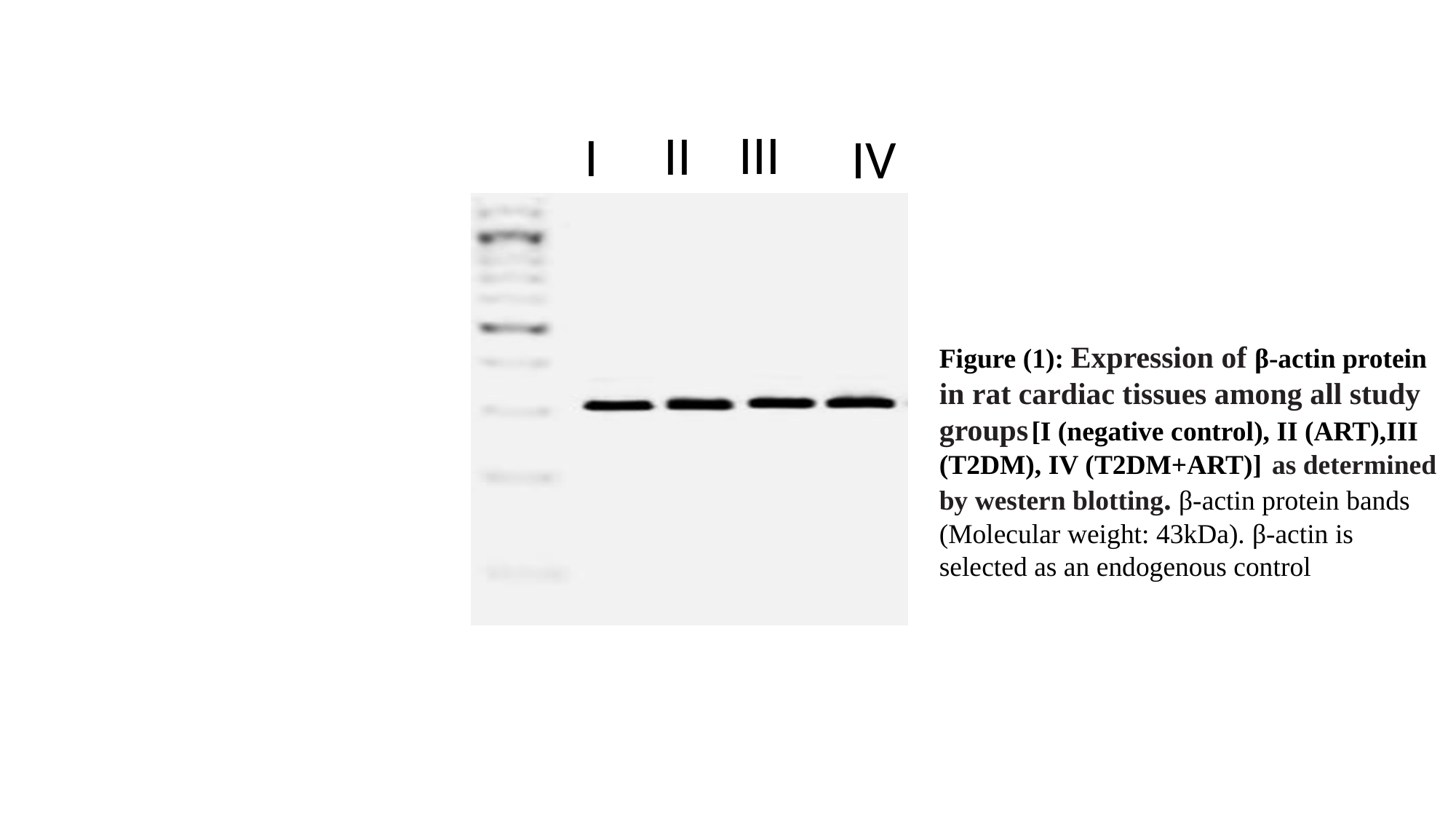

III
II
I
IV
Figure (1): Expression of β-actin protein in rat cardiac tissues among all study groups [I (negative control), II (ART),III (T2DM), IV (T2DM+ART)] as determined by western blotting. β-actin protein bands (Molecular weight: 43kDa). β-actin is selected as an endogenous control
